# The crystal structure of (C_2_H_9_N_2_)_2_[Zn_3_(HPO_3_)_4_], a three-dimensional zincophosphite framework containing 16-membered rings templated by the unsymmetrical dimethyl hydrazinium cation

**DOI:** 10.1107/S2056989017005758

**Published:** 2017-04-28

**Authors:** Judita Katinaitė, William T. A. Harrison

**Affiliations:** a Department of Chemistry, University of Aberdeen, Meston Walk, Aberdeen AB24 3UE, Scotland

**Keywords:** crystal structure, zinc phosphite, unsymmetrical dimethyl hydrazine, open framework

## Abstract

The solution-mediated synthesis and crystal structure of 1,1-di­methyl­hydrazinium tetra­phoshonoatotrizincate, (C_2_H_9_N_2_)_2_[Zn_3_(HPO_3_)_4_], are described. The anionic [Zn_3_(HPO_3_)_4_]^2−^ framework is built up from alternating ZnO_4_ tetra­hedra and HPO_3_ pseudo-pyramids to generate a three-dimensional 4,3-net encapsulating the C_2_H_9_N_2_^+^ cations. The organic cations, which are protonated at their central N atoms, occupy pores delineated by large 16-membered polyhedral rings and inter­act with the framework by way of N—H⋯O hydrogen bonds and possible C—H⋯O inter­actions. One of the zinc ions lies on a crystallographic twofold rotation axis and all the other atoms lie on general positions. The crystal studied was found to be rotationally twinned about the [001] axis in reciprocal space in a 0.585 (5):0.415 (5) ratio.

## Abstract 2

The title open framework contains 16-ring pores templated by pairs of side-by-side unsymmetrical dimethyl hydrazinium cations.

## Chemical context   

Organically templated zinc phosphites are now a well-established family of open frameworks (e.g.: Phillips *et al.*, 2002 ▸; Luo *et al.*, 2010 ▸; Wang *et al.*, 2011 ▸; Dong *et al.*, 2015 ▸; Huang *et al.*, 2017 ▸). As part of our occasional studies in this area (Harrison & McNamee, 2010 ▸), we now describe the synthesis and structure of the title compound, (I), which represents the first example of a protonated unsymmetrical dimethyl hydrazine (C_2_H_8_N_2_ or UDMH is the neutral mol­ecule and C_2_H_9_N_2_^+^ is the cation) acting as a templating agent for an inorganic open framework. So far as we are aware, the only crystal structures containing C_2_H_9_N_2_^+^ that have been reported previously are mol­ecular salts with different simple counter-ions (Katinaitė & Harrison, 2016 ▸, and references therein).
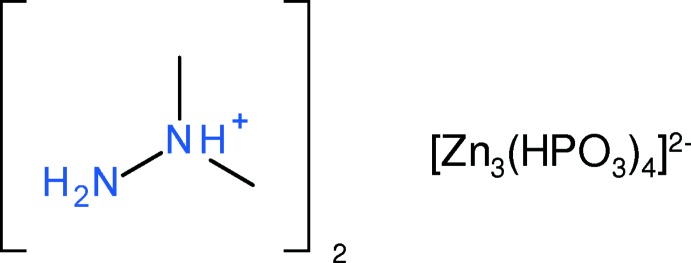


## Structural commentary   

**Figure 1:**
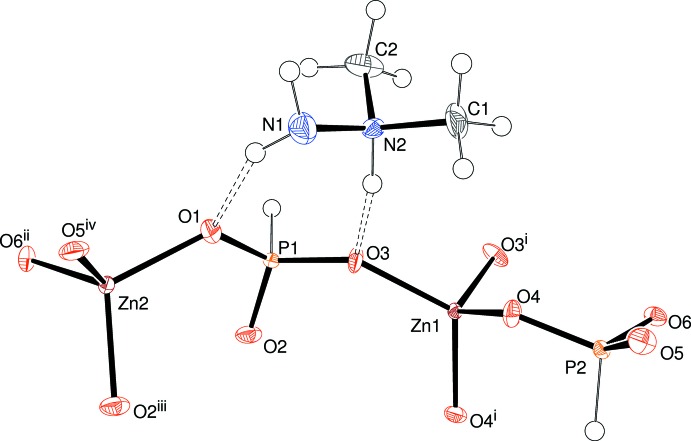
The asymmetric unit of (I) expanded to show the zinc coordination polyhedra (50% displacement ellipsoids). For symmetry codes, see Table 1 ▸.

The asymmetric unit of (I) comprises two zinc cations (Zn1 with site symmetry 2 and Zn2 on a general position), two HPO_3_^2−^ hydrogen phosphite groups and one C_2_H_9_N_2_^+^ cation (Fig. 1 ▸). Both zinc ions adopt their usual tetra­hedral coord­ination geometries (Table 1 ▸) to four nearby O atoms with mean Zn—O separations of 1.942 and 1.945 Å for Zn1 and Zn2, respectively. The range of O—Zn—O bond angles for Zn1 of 100.0 (2)–121.0 (2)° indicates considerable distortion from the ideal tetra­hedral value of 109.5°; the spread of values for Zn2 of 99.8 (2)–115.1 (2)° is somewhat smaller. Bond-valence-sum values (in valence units; Brown & Altermatt, 1985 ▸) for Zn1 and Zn2 of 2.11 and 2.09, respectively, are in adequate agreement with the expected values of 2.00.

Both phospho­rus atoms in (I) display their expected HPO_3_ pseudo-tetra­hedral geometries with mean P—O distances (1.517 Å for P1 and 1.516 Å for P2) and O—P—O angles (112.7° for P1 and 112.6° for P2) that are consistent with previous results (Dong *et al.*, 2015 ▸). P1 deviates from its pyramid of attached O atoms by 0.418 (4) and the equivalent deviation for P2 is 0.420 (3) Å.

The structure of (I) is completed by the charge-balancing C_2_H_9_N_2_^+^ cation, which is protonated at the central (methyl­ated) N2 atom, as is most commonly seen for this species (Katinaitė & Harrison, 2016 ▸). The C—N and N—N bond lengths are indistinguishable and N2 deviates from the plane of N1, C1 and C2 by 0.434 (8) Å.

In the extended framework structure of (I), the zinc- and phospho­rus-centred building units strictly alternate: every O atom forms a Zn—O—P bridge (mean angle = 131.4°), thus there are no ‘dangling’ Zn—OH_2_, P=O or P—OH bonds as found in some zincophosphite frameworks (Shi *et al.*, 2004 ▸; Liu *et al.*, 2008 ▸), which is fully consistent with the 3:4 Zn:P stoichiometry of the anionic [Zn_3_(HPO_3_)_4_]^2−^ component of the structure (Harrison & McNamee, 2010 ▸). In addition, there are no Zn—N bonds (direct metal-to-template links) in (I); compare Kirkpatrick & Harrison (2004 ▸), Lin *et al.* (2004 ▸) and Harrison (2006 ▸).

The polyhedral connectivity in (I) can be broken down as follows: the Zn2, P1 and P2 polyhedra form four-ring (*i.e*.: a loop of two Zn atoms and two P atoms) chains, with the zinc atoms as the linking nodes, which propagate alternately in the [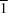
10] and [110] directions with respect to the *c*-axis direction. Atom Zn1 serves to link these criss-cross chains into a three-dimensional open framework. If the template is omitted, a *PLATON* (Spek, 2009 ▸) analysis indicates that 878 Å^3^ (43.3%) of the unit cell is ‘empty space’ and the ‘framework density’ (FD) (number of Zn and P atoms per 1000 Å^3^; Brunner & Meier, 1989 ▸) of (I) is 13.8. This low FD is comparable to that of the unusual open-framework MAPSO-46, which contains Mg, Al, P and Si as its tetra­hedral framework atoms (Bennett & Marcus, 1988 ▸). When the template is included in the calculation, *PLATON* indicates no free space, suggesting that the template is a ‘snug fit’ within the inorganic framework of (I).

**Figure 2:**
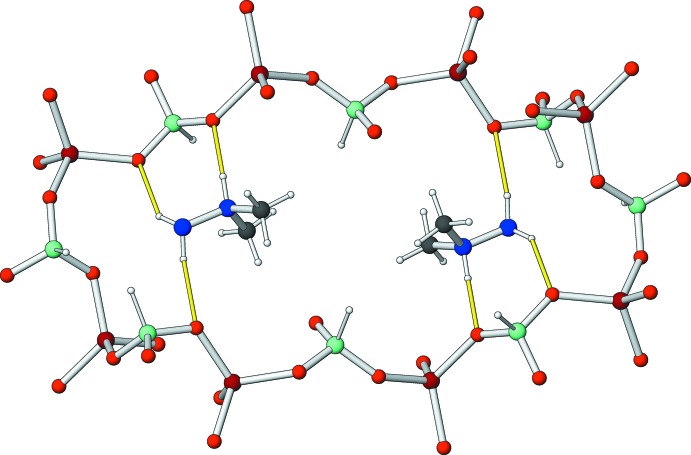
Fragment of (I) showing a 16-ring channel occupied side-by-side by two C_2_H_9_N_2_^+^ template cations.

In the extended structure, large 16-ring pores (Figs. 2 ▸ and 3 ▸) are apparent in the framework, which alternately propagate in [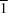
10] and [110] with respect to the *c*-axis direction. Measured atom-to-atom, the 16-ring has a dimension of ∼5.7 × 14.6 Å. Pairs of template cations lie roughly in the plane of the 16-rings and inter­act with framework oxygen atoms by way of N—H⋯O hydrogen bonds (Table 2 ▸). It is notable that the H⋯O separation for the charge-assisted N2^+^—H3*N*⋯O3 bond is much shorter than the H⋯O separations for the terminal N1H_2_ grouping. Within the asymmetric unit, an 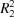
(7) loop is apparent (Fig. 1 ▸). Possible weak C—H⋯O inter­actions (Table 2 ▸) consolidate the structure.

## Database survey   

A survey of of the Cambridge Structural Database (Groom *et al.*, 2016 ▸: updated to April 2017) for organically templated zinc phosphite frameworks (those containing a Zn—O—P—H fragment) revealed 172 matches.

## Synthesis and crystallization   

Caution! UDMH is toxic, potentially carcinogenic and may form explosive mixtures with oxidizing agents: all appropriate safety precautions should be taken when handling it. Zinc oxide (1.63 g), phospho­rus acid (1.64 g) and 20 ml of a 1.0 *M* aqueous UDMH solution were mixed in a 1:1:1 molar ratio in a sealed PTFE bottle and heated to 353 K for 24 h and then cooled to room temperature over a few hours. Product recovery by vacuum filtration yielded some colourless blocks of (I) accompanied by an unidentified white powder.

## Refinement   

Crystal data, data collection and structure refinement details are summarized in Table 3 ▸. The N-bound H atoms were located in difference maps, relocated to idealized locations (N—H = 0.91–1.00 Å) and refined as riding atoms. The other hydrogen atoms were placed geometrically (P—H = 1.32, C—H = 0.98 Å) and refined as riding atoms. The constraint *U*_iso_(H) = 1.2*U*_eq_(carrier) or 1.5*U*_eq_(methyl carrier) was applied in all cases. The methyl groups were allowed to rotate, but not to tip, to best fit the electron density. The crystal chosen for data collection was found to be rotationally twinned about the [001] axis in reciprocal space in a 0.585 (5):0.415 (5) ratio.

## Supplementary Material

Crystal structure: contains datablock(s) I, global. DOI: 10.1107/S2056989017005758/sj5526sup1.cif

Structure factors: contains datablock(s) I. DOI: 10.1107/S2056989017005758/sj5526Isup2.hkl

CCDC reference: 1544227

Additional supporting information:  crystallographic information; 3D view; checkCIF report

## Figures and Tables

**Figure 3:**
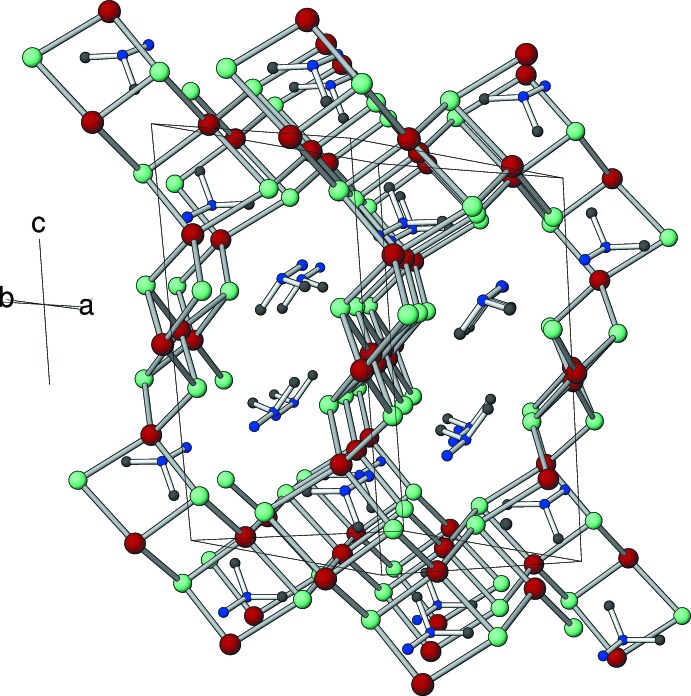
The unit-cell packing in (I) viewed approximately along [110] with the framework represented topologically (*i.e.* as Zn—P links).

**Table 1:** Selected geometric parameters (Å, °)

Zn1—O4	1.938 (5)	P1—O2	1.504 (5)
Zn1—O4^i^	1.938 (5)	P1—O1	1.515 (6)
Zn2—O5^ii^	1.936 (6)	P1—O3	1.533 (5)
Zn2—O2^iii^	1.943 (5)	P2—O5	1.500 (6)
Zn2—O6^iv^	1.946 (5)	P2—O6	1.520 (5)
Zn2—O1	1.954 (6)	P2—O4	1.529 (5)
			
P1—O1—Zn2	128.0 (3)	P2—O4—Zn1	123.6 (3)
P1—O2—Zn2^iii^	140.3 (3)	P2—O5—Zn2^v^	138.4 (4)
P1—O3—Zn1	137.2 (3)	P2—O6—Zn2^vi^	120.8 (3)

Symmetry codes: (i) 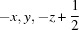; (ii) 
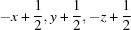
; (iii) 
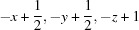
; (iv) 
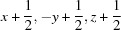
; (v) 
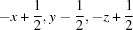
; (vi) 
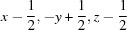
.

**Table 2:** Hydrogen-bond geometry (Å, °)

*D*—H⋯*A*	*D*—H	H⋯*A*	*D*⋯*A*	*D*—H⋯*A*
N1—H1*N*⋯O1	0.91	2.34	3.130 (9)	146
N1—H2*N*⋯O6^vii^	0.91	2.35	3.133 (9)	144
N2—H3*N*⋯O3	1.00	1.79	2.762 (8)	163
C1—H1*A*⋯O1^v^	0.98	2.50	3.474 (11)	173
C1—H1*C*⋯O5^viii^	0.98	2.50	3.295 (11)	138
C2—H2*C*⋯O2^ix^	0.98	2.43	3.355 (9)	157

Symmetry codes: (v) 
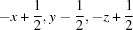
; (vii) 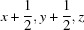; (viii) 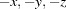; (ix) 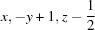.

**Table 3:** Experimental details

Crystal data	
Chemical formula	(C_2_H_9_N_2_)_2_[Zn_3_(HPO_3_)_4_]
*M*_r_	638.24
Crystal system, space group	Monoclinic, *C*2/*c*
Temperature (K)	100
*a*, *b*, *c* (Å)	15.1154 (5), 8.7269 (3), 16.1675 (6)
β (°)	108.156 (1)
*V* (Å^3^)	2026.48 (12)
*Z*	4
Radiation type	Mo *K*α
μ (mm^−1^)	3.90
Crystal size (mm)	0.19 × 0.11 × 0.05

Data collection	
Diffractometer	Rigaku Mercury CCD
Absorption correction	Multi-scan (*SADABS*; Sheldrick, 2004 ▸)
*T*_min_, *T*_max_	0.527, 1.000
No. of measured, independent and observed [*I* > 2σ(*I*)] reflections	2273, 2273, 2169
(sin θ/λ)_max_ (Å^−1^)	0.650

Refinement	
*R*[*F* ^2^ > 2σ(*F* ^2^)], *wR*(*F* ^2^), *S*	0.068, 0.239, 1.22
No. of reflections	2273
No. of parameters	127
H-atom treatment	H-atom parameters constrained
Δρ_max_, Δρ_min_ (e Å^−3^)	1.51, −1.24

Computer programs: *CrysAlis PRO* (Rigaku, 2015 ▸), *SHELXS97* (Sheldrick, 2008 ▸), *SHELXL2014* (Sheldrick, 2015 ▸), *ORTEP-3 for Windows* (Farrugia, 2012 ▸), *ATOMS* (Shape Software, 2005 ▸) and *publCIF* (Westrip, 2010 ▸).

## supplementary crystallographic information

### Crystal data

**Table:**

(C_2_H_9_N_2_)_2_[Zn_3_(HPO_3_)_4_]	*F*(000) = 1280
*M**_r_* = 638.24	*D*_x_ = 2.092 Mg m^−^^3^
Monoclinic, *C*2/*c*	Mo *K*α radiation, λ = 0.71073 Å
*a* = 15.1154 (5) Å	Cell parameters from 6272 reflections
*b* = 8.7269 (3) Å	θ = 2.6–27.6°
*c* = 16.1675 (6) Å	µ = 3.90 mm^−^^1^
β = 108.156 (1)°	*T* = 100 K
*V* = 2026.48 (12) Å^3^	Block, colourless
*Z* = 4	0.19 × 0.11 × 0.05 mm

### Data collection

**Table:**

Rigaku Mercury CCD diffractometer	2169 reflections with *I* > 2σ(*I*)
ω scans	θ_max_ = 27.5°, θ_min_ = 2.7°
Absorption correction: multi-scan (SADABS; Sheldrick, 2004)	*h* = −19→18
*T*_min_ = 0.527, *T*_max_ = 1.000	*k* = −11→11
2273 measured reflections	*l* = −11→20
2273 independent reflections	

### Refinement

**Table:**

Refinement on *F*^2^	Hydrogen site location: mixed
Least-squares matrix: full	H-atom parameters constrained
*R*[*F*^2^ > 2σ(*F*^2^)] = 0.068	*w* = 1/[σ^2^(*F*_o_^2^) + (0.1587*P*)^2^ + 13.6003*P*] where *P* = (*F*_o_^2^ + 2*F*_c_^2^)/3
*wR*(*F*^2^) = 0.239	(Δ/σ)_max_ < 0.001
*S* = 1.22	Δρ_max_ = 1.51 e Å^−^^3^
2273 reflections	Δρ_min_ = −1.24 e Å^−^^3^
127 parameters	Extinction correction: SHELXL2014 (Sheldrick, 2015), Fc^*^=kFc[1+0.001xFc^2^λ^3^/sin(2θ)]^-1/4^
0 restraints	Extinction coefficient: 0.011 (2)
Primary atom site location: structure-invariant direct methods	

### Special details

**Table:**

Geometry. All esds (except the esd in the dihedral angle between two l.s. planes) are estimated using the full covariance matrix. The cell esds are taken into account individually in the estimation of esds in distances, angles and torsion angles; correlations between esds in cell parameters are only used when they are defined by crystal symmetry. An approximate (isotropic) treatment of cell esds is used for estimating esds involving l.s. planes.
Refinement. Refined as a 2-component twin with components rotated about (001) in reciprocal space

### Fractional atomic coordinates and isotropic or equivalent isotropic displacement parameters (Å^2^)

**Table:**

	*x*	*y*	*z*	*U*_iso_*/*U*_eq_	
Zn1	0.0000	0.16565 (13)	0.2500	0.0076 (4)	
Zn2	0.36570 (6)	0.37073 (9)	0.48484 (5)	0.0096 (4)	
P1	0.15444 (13)	0.3885 (2)	0.37817 (11)	0.0091 (5)	
H1	0.1147	0.5248	0.3662	0.011*	
P2	−0.01597 (13)	−0.0917 (2)	0.11747 (11)	0.0088 (5)	
H2	−0.0253	−0.1970	0.1728	0.011*	
O1	0.2557 (4)	0.4152 (7)	0.3858 (3)	0.0193 (12)	
O2	0.1393 (5)	0.3246 (6)	0.4592 (3)	0.0180 (12)	
O3	0.1081 (4)	0.2975 (7)	0.2948 (3)	0.0160 (11)	
O4	0.0428 (4)	0.0362 (6)	0.1728 (3)	0.0146 (11)	
O5	0.0321 (4)	−0.1690 (6)	0.0604 (4)	0.0169 (12)	
O6	−0.1139 (4)	−0.0379 (6)	0.0684 (3)	0.0124 (10)	
C1	0.1591 (7)	0.2877 (11)	0.0880 (5)	0.028 (2)	
H1A	0.1821	0.1825	0.1002	0.043*	
H1B	0.1847	0.3339	0.0452	0.043*	
H1C	0.0909	0.2867	0.0649	0.043*	
C2	0.1529 (8)	0.5365 (10)	0.1562 (6)	0.029 (2)	
H2A	0.1818	0.5963	0.2091	0.043*	
H2B	0.0852	0.5358	0.1439	0.043*	
H2C	0.1683	0.5827	0.1072	0.043*	
N1	0.2898 (6)	0.3663 (9)	0.2063 (5)	0.0249 (17)	
H1N	0.3067	0.3880	0.2642	0.030*	
H2N	0.3170	0.4346	0.1793	0.030*	
N2	0.1884 (5)	0.3781 (7)	0.1691 (4)	0.0132 (13)	
H3N	0.1608	0.3280	0.2110	0.016*	

### Atomic displacement parameters (Å^2^)

**Table:**

	*U*^11^	*U*^22^	*U*^33^	*U*^12^	*U*^13^	*U*^23^
Zn1	0.0064 (7)	0.0080 (6)	0.0076 (6)	0.000	0.0008 (4)	0.000
Zn2	0.0093 (6)	0.0109 (5)	0.0078 (5)	−0.0032 (3)	0.0015 (4)	0.0003 (3)
P1	0.0106 (10)	0.0085 (8)	0.0076 (8)	−0.0011 (6)	0.0021 (7)	0.0010 (6)
P2	0.0085 (9)	0.0093 (8)	0.0077 (8)	0.0008 (6)	0.0014 (7)	0.0001 (6)
O1	0.020 (3)	0.024 (3)	0.012 (2)	−0.006 (2)	0.001 (2)	0.002 (2)
O2	0.033 (3)	0.009 (2)	0.013 (2)	−0.002 (2)	0.009 (2)	0.0000 (18)
O3	0.013 (3)	0.023 (3)	0.012 (2)	−0.010 (2)	0.004 (2)	−0.006 (2)
O4	0.015 (3)	0.014 (2)	0.014 (2)	0.000 (2)	0.002 (2)	−0.006 (2)
O5	0.025 (3)	0.012 (2)	0.016 (2)	0.005 (2)	0.009 (2)	−0.0021 (19)
O6	0.008 (3)	0.016 (2)	0.013 (2)	0.000 (2)	0.0020 (19)	0.004 (2)
C1	0.039 (5)	0.024 (4)	0.015 (3)	−0.003 (4)	−0.002 (4)	−0.008 (3)
C2	0.048 (6)	0.020 (4)	0.024 (4)	0.005 (4)	0.020 (4)	0.006 (3)
N1	0.019 (4)	0.034 (4)	0.020 (3)	−0.004 (3)	0.004 (3)	0.002 (3)
N2	0.017 (4)	0.013 (3)	0.011 (3)	−0.004 (2)	0.008 (3)	−0.001 (2)

### Geometric parameters (Å, º)

**Table:**

Zn1—O4	1.938 (5)	O2—Zn2^iii^	1.943 (5)
Zn1—O4^i^	1.938 (5)	O5—Zn2^v^	1.936 (6)
Zn1—O3^i^	1.945 (5)	O6—Zn2^vi^	1.946 (5)
Zn1—O3	1.945 (5)	C1—N2	1.475 (9)
Zn2—O5^ii^	1.936 (6)	C1—H1A	0.9800
Zn2—O2^iii^	1.943 (5)	C1—H1B	0.9800
Zn2—O6^iv^	1.946 (5)	C1—H1C	0.9800
Zn2—O1	1.954 (6)	C2—N2	1.474 (10)
P1—O2	1.504 (5)	C2—H2A	0.9800
P1—O1	1.515 (6)	C2—H2B	0.9800
P1—O3	1.533 (5)	C2—H2C	0.9800
P1—H1	1.3200	N1—N2	1.465 (10)
P2—O5	1.500 (6)	N1—H1N	0.9100
P2—O6	1.520 (5)	N1—H2N	0.9100
P2—O4	1.529 (5)	N2—H3N	1.0000
P2—H2	1.3200		
			
O4—Zn1—O4^i^	108.7 (3)	P1—O3—Zn1	137.2 (3)
O4—Zn1—O3^i^	121.0 (2)	P2—O4—Zn1	123.6 (3)
O4^i^—Zn1—O3^i^	100.0 (2)	P2—O5—Zn2^v^	138.4 (4)
O4—Zn1—O3	100.0 (2)	P2—O6—Zn2^vi^	120.8 (3)
O4^i^—Zn1—O3	121.0 (2)	N2—C1—H1A	109.5
O3^i^—Zn1—O3	107.4 (4)	N2—C1—H1B	109.5
O5^ii^—Zn2—O2^iii^	99.8 (2)	H1A—C1—H1B	109.5
O5^ii^—Zn2—O6^iv^	115.1 (2)	N2—C1—H1C	109.5
O2^iii^—Zn2—O6^iv^	110.8 (2)	H1A—C1—H1C	109.5
O5^ii^—Zn2—O1	107.5 (3)	H1B—C1—H1C	109.5
O2^iii^—Zn2—O1	114.1 (3)	N2—C2—H2A	109.5
O6^iv^—Zn2—O1	109.3 (2)	N2—C2—H2B	109.5
O2—P1—O1	114.2 (3)	H2A—C2—H2B	109.5
O2—P1—O3	115.0 (3)	N2—C2—H2C	109.5
O1—P1—O3	108.9 (3)	H2A—C2—H2C	109.5
O2—P1—H1	106.0	H2B—C2—H2C	109.5
O1—P1—H1	106.0	N2—N1—H1N	109.3
O3—P1—H1	106.0	N2—N1—H2N	109.2
O5—P2—O6	113.4 (3)	H1N—N1—H2N	109.5
O5—P2—O4	112.6 (3)	N1—N2—C2	114.3 (7)
O6—P2—O4	111.9 (3)	N1—N2—C1	108.3 (6)
O5—P2—H2	106.1	C2—N2—C1	112.4 (7)
O6—P2—H2	106.1	N1—N2—H3N	107.2
O4—P2—H2	106.1	C2—N2—H3N	107.2
P1—O1—Zn2	128.0 (3)	C1—N2—H3N	107.2
P1—O2—Zn2^iii^	140.3 (3)		
			
O2—P1—O1—Zn2	2.2 (6)	O5—P2—O4—Zn1	177.2 (3)
O3—P1—O1—Zn2	−127.8 (4)	O6—P2—O4—Zn1	48.1 (4)
O1—P1—O2—Zn2^iii^	−86.8 (7)	O6—P2—O5—Zn2^v^	110.4 (6)
O3—P1—O2—Zn2^iii^	40.2 (8)	O4—P2—O5—Zn2^v^	−18.0 (7)
O2—P1—O3—Zn1	28.0 (7)	O5—P2—O6—Zn2^vi^	−68.4 (4)
O1—P1—O3—Zn1	157.6 (5)	O4—P2—O6—Zn2^vi^	60.3 (4)

Symmetry codes: (i) −*x*, *y*, −*z*+1/2; (ii) −*x*+1/2, *y*+1/2, −*z*+1/2; (iii) −*x*+1/2, −*y*+1/2, −*z*+1; (iv) *x*+1/2, −*y*+1/2, *z*+1/2; (v) −*x*+1/2, *y*−1/2, −*z*+1/2; (vi) *x*−1/2, −*y*+1/2, *z*−1/2.

### Hydrogen-bond geometry (Å, º)

**Table:**

*D*—H···*A*	*D*—H	H···*A*	*D*···*A*	*D*—H···*A*
N1—H1*N*···O1	0.91	2.34	3.130 (9)	146
N1—H2*N*···O6^vii^	0.91	2.35	3.133 (9)	144
N2—H3*N*···O3	1.00	1.79	2.762 (8)	163
C1—H1*A*···O1^v^	0.98	2.50	3.474 (11)	173
C1—H1*C*···O5^viii^	0.98	2.50	3.295 (11)	138
C2—H2*C*···O2^ix^	0.98	2.43	3.355 (9)	157

Symmetry codes: (v) −*x*+1/2, *y*−1/2, −*z*+1/2; (vii) *x*+1/2, *y*+1/2, *z*; (viii) −*x*, −*y*, −*z*; (ix) *x*, −*y*+1, *z*−1/2.
